# *JC Polyomavirus*, progressive multifocal leukoencephalopathy and immune reconstitution inflammatory syndrome: a review

**DOI:** 10.1186/s12981-020-00293-0

**Published:** 2020-07-06

**Authors:** Vijay Harypursat, Yihong Zhou, Shengquan Tang, Yaokai Chen

**Affiliations:** Division of Infectious Diseases, Chongqing Public Health Medical Center, 109 Baoyu Road, Geleshan Town, Shapingba District, Chongqing, 400036 People’s Republic of China

**Keywords:** Immunosuppression, Oligodendrocyte, Monoclonal antibodies, Immunocompromised state

## Abstract

The human neurotropic virus *JC Polyomavirus*, a member of the *Polyomaviridae* family, is the opportunistic infectious agent causing progressive multifocal leukoencephalopathy, typically in immunocompromised individuals. The spectrum of underlying reasons for the systemic immunosuppression that permits JCV infection in the central nervous system has evolved over the past 2 decades, and therapeutic immunosuppression arousing JCV infection in the brain has become increasingly prominent as a trigger for PML. Effective immune restoration subsequent to human immunodeficiency virus-related suppression is now recognized as a cause for unexpected deterioration of symptoms in patients with PML, secondary to a rebound inflammatory phenomenon called immune reconstitution inflammatory syndrome, resulting in significantly increased morbidity and mortality in a disease already infamous for its lethality. This review addresses current knowledge regarding *JC Polyomavirus*, progressive multifocal leukoencephalopathy, progressive multifocal leukoencephalopathy-related immune reconstitution inflammatory syndrome, and the immunocompromised states that incite *JC Polyomavirus* central nervous system infection, and discusses prospects for the future management of these conditions.

## Introduction

*JC Polyomavirus* (JCV, or JCPyV) infection is ubiquitous in humans [1–5]. Infection is asymptomatic and occurs in childhood, adolescence, and adulthood [6–8]. The organism establishes latency in renal and other tissues, and may remain in an inactive latent state in these tissues for the rest of the host’s natural life without causing disease. Disease processes that result in profound systemic immunosuppression may initiate a poorly understood sequence of events that cause re-activation of JCV in the central nervous system (CNS), with subsequent establishment of active opportunistic JCV infection in tissues of the CNS [7]. Active JCV infection in the CNS has a high morbidity and mortality. Before the advent of highly active anti-retroviral therapy (HAART) for acquired immunodeficiency syndrome (AIDS) (prior to 1996), the most common cause for the immunosuppression causing JCV-related CNS pathology was AIDS, and the most common manifestation of JCV CNS involvement was progressive multifocal leukoencephalopathy (PML) [9].

While the effectiveness of HAART in restoring immune competence in AIDS patients has resulted in a substantial decrease in the prevalence of human immunodeficiency virus (HIV)-related PML [9], it has also resulted in a corresponding increase in the incidence of immune reconstitution inflammatory syndrome (IRIS) in these patients [10], which in itself has morbidity and mortality implications, particularly if not diagnosed early. It is now recognized that IRIS may occur following restoration of immune competence after systemic immunocompromise from several causes, and may also be associated with various different opportunistic infections [11]. CNS-IRIS presents a diagnostic and therapeutic challenge to clinicians, as clinical signs and symptoms, and therapeutic options for the different types of CNS-IRIS, differ depending on the patient’s underlying immunological state, and on their respective underlying opportunistic infections [10]. It is now also universally acknowledged that JCV causes pathology other than PML in the CNS [9, 12, 13]. Furthermore, new information regarding the pathogenesis of PML may refine the diagnostic and therapeutic options available to clinicians for the treatment of PML in the future. In this review, we examine current information regarding JCV, and the pathogenesis and treatment of PML and PML-IRIS in immunocompromised patients.

## Human *Polyomavirus JC*

JCV is a ubiquitous, species-specific DNA virus that has been associated with the human race for most of human evolution, and infects more than 70% of the human population [1–5], depending on age and country. While some viruses have been introduced to the human population relatively recently in our evolution (HIV, Ebola), JCV has been a constant companion of the mammals that evolved into humans for millions of years [14]. JCV age-specific prevalence rises from around 16% in children 1–5 years of age, to 34% by age 21, and increases to 51% over the age of 50 [9, 15]. In an immunocompetent human host, primary JCV infection is asymptomatic, and JCV subsequently locates to certain tissues e.g., bone marrow, brain, tonsils, renal cells, and lungs [6–8], and remains latent in these tissues of healthy people. This dormant JCV variant is termed the ‘archetype’ strain of JCV, as it is thought that this is the original variant from which all other JCV variants evolved [8]. Archetypal JCV does not replicate effectively in macroglial cells, and undergoes genetic re-arrangement in the noncoding regulatory region of its genome to transform into the neurotropic ‘prototype’ JCV that is associated with oligodendrocytic infection and CNS disease [7]. This prototype variant contains rearrangements to its genome which include duplications, tandem repeats, insertions and deletions, and is the form of JCV most often found in the cerebrospinal fluid (CSF) and brain tissue from patients with PML [8]. In circumstances of sustained immune suppression, productive infection by the prototype variant of JCV occurs in the CNS. It is unknown whether the prototype variant originates in the brain or in peripheral tissues, or indeed both in the brain and in peripheral tissues [16]. How the immune system normally prohibits JC viral replication and spread into the CNS, and how compromised immunity permits JCV to spread throughout the CNS white matter has not been precisely elucidated [17]. Although PML usually affects individuals with profound cellular immunosuppression, it has also rarely been diagnosed in patients with no initial clinically apparent immunosuppression [8, 18]. In these patients, a potential diagnosis of idiopathic CD4+ lymphocytopenia should be considered. The putative role of *JC Polyomavirus* in animal and human oncogenesis is beyond the scope of this review, and is discussed elsewhere [19, 20].

The prototype JCV variant displays strong tropism for glial cells, and replication of this strain in macroglial cells, predominantly oligodendrocytes, precipitates demyelination of white matter in the CNS, manifesting clinically as PML [21]. Prototype JCV can also infect astrocytes, and cells of the choroid plexus and leptomeninges, which are critical components of the blood–brain barrier [22], and is now known to infect granule cells in the cerebellum, causing JCV granule cell neuronopathy, cortical pyramidal neurons, causing JCV encephalopathy [12], and the leptomeninges, which causes JCV meningitis, meningo-encephalitis, and the meningeal syndrome [13]. JCV likely reaches the sites of initial intrathecal infection via hematogenous spread. Here they infect perivascular oligodendrocytes. Subsequent viral replication causes lysis of the infected oligodendrocyte, release of progeny viral particles, and loss of a small segment of myelin sheath along an axon [23]. Progeny viral particles from the lysed oligodendrocyte then infect adjacent oligodendrocytes along the involved axon, as well as oligodendrocytes ensheathing segments of adjacent axons. In this manner, miniscule initial cerebral and cerebellar perivascular foci of demyelination establish, extend, expand and coalesce, resulting in the familiar magnetic resonance fluid-attenuated inversion recovery (MRI/FLAIR) image of advanced PML, showing vast areas of demyelination in the white, and sometimes gray matter of the brain [23]. Active JCV genetic mutation in the brain causes multiple JCV variants to co-exist in the CNS of PML patients, containing mutations throughout the viral genome, indicating a dynamic evolution of the viral genome within the CNS of patients with PML [16]. There is no evidence that the presence of JCV antibodies offer any protection from current or future infection, or from virus reactivation [24]. The cellular immune response is most important in controlling JCV reactivation, and for the prevention of JCV-related CNS disease.

## Progressive multifocal leukoencephalopathy

PML tends not to occur in immunologically healthy people, and develops after JCV infection primarily in oligodendrocytes, with subsequent demyelination of subcortical white matter in the CNS by lysis of oligodendrocytes [25]. Clinical features of PML comprise motor weakness (monoparesis or hemiparesis), cognitive dysfunction, appendicular or gait ataxia, visual symptoms (hemianopsia, diplopia), and speech disturbances [26]. Headaches, seizures and sensory loss are less frequently present. Of those patients that develop HIV-PML, just over 50% will die within 24 months [12, 27]. Significant morbidity accompanies those who survive. Brain MRI in PML classically shows hypointense lesions on T1-weighted images, and hyperintensity on T2-weighted and FLAIR images [28]. In computed tomography (CT) scans, brain lesions appear as asymmetric multifocal areas of hypodensity. Generally, multiple bilateral asymmetrical brain lesions are present, and localize to subcortical hemispheric white matter or the cerebellar peduncles. Edema, mass effect, and contrast enhancement are usually absent in classical PML brain radiology. Some PML lesions may be found in gray matter structures where myelinated fibers reside e.g., basal ganglia and thalamus [8, 28]. Currently, the most common causes for the underlying immunosuppression resulting in the development of PML is AIDS, followed by hematological malignancies, and immunomodulatory therapies used in a subgroup of multiple sclerosis (MS) patients [29].

Definitive diagnosis of PML is established by brain biopsy via in situ hybridization of JCV deoxyribonucleic acid (DNA) or immunohistochemical staining for JCV DNA, or by detection of JCV DNA in CSF by polymerase chain reaction (PCR). PCR for JCV DNA in the CSF of PML patients is quite specific and sensitive (> 95%) in patients not on HAART. However, the sensitivity of CSF JCV PCR in patients on HAART decreases to less than 60%, due to the relatively low CSF JCV viral loads in PML patients whose suppressed immune systems are resuscitated by HAART [30]. The accuracy of JCV DNA detection by PCR is dependent on the detection limits of different JCV DNA PCR tests used at particular laboratories. Some laboratories use techniques that have a lower limit of DNA detection of 20 DNA copies/mL, while a large number of laboratories use tests that have a higher detection limit of 200 DNA copies/mL. This difference in detection thresholds can lead to a significant number of false negative results in laboratories with higher DNA detection limits, and thus confers lower specificity for JCV DNA testing in these particular laboratories. It is also reasonable to repeat the CSF DNA PCR 24–48 h later in a patient with a strong suspicion of JCV CNS infection, if the initial sample result is negative. Interestingly, a recently published study indicates that it is possible to lower the detection limit for real time JCV DNA PCR by viral enrichment using ultrafiltration, when extremely low copy numbers of JCV are released into the CSF (as in patients on HAART), or when brain biopsy is not feasible in patients on HAART [31]. Brain biopsy is associated with significant risk of fatal complications, and has a high index of morbidity [32]. Histology of PML-affected brain tissue shows productive infection of macroglial cells, with the presence of the histological ‘classical triad’ of microscopic features, comprising subcortical demyelination, bizarre giant astrocytes, and large oligodendrocytes with enlarged nuclei harboring a large number of intranuclear eosinophilic inclusion bodies, which represents active viral replication [19]. Some affected areas may reveal reactive astrogliosis, with giant bizarre multinucleated astrocytes present. A diagnosis of “probable” or “possible” PML may be made in the presence of typical PML clinical and radiological findings only, in the absence of positive observation of JCV DNA in CSF or brain tissue, if other causes have been ruled out [33].

A specific antiviral agent against JCV to treat PML in HIV immunosuppressed patients does not exist at the present time [27], and primary treatment focuses on restoration of immune system fecundity by initiating HAART expeditiously. Optimal immune reconstitution to control JCV, without causing CNS-damaging IRIS is currently the most practical approach to treat PML [34]. PML survival has increased from 1–30% in the pre-ART era, to 38–62% after the introduction of HAART [8, 35], with approximately 50% of HIV-PML patients experiencing an arrest of disease progression after initiating HAART [10]. Mirtazapine, a serotonin receptor antagonist, has been shown in case reports to produce favorable outcomes in some cases of PML [36, 37]. However, convincingly strong supporting evidence for mirtazapine efficacy in the treatment of PML is lacking in the literature at this time. Mefloquine, an anti-malaria agent, has in the past been shown to inhibit JCV replication in vitro [38]. However, a clinical study investigating the use of Mefloquine for the treatment of PML was terminated early upon recommendation of the study data safety committee, after conditional power calculations suggested a very small probability that continuation of the study would demonstrate any difference in the primary endpoint [39]. The antiviral agent, cidofovir, and a chemotherapeutic agent, cytarabine, showed initial promise in the treatment of PML in in vitro and clinical testing; however, neither of these agents have demonstrated significant survival benefit when studied under controlled human clinical trial conditions [40, 41].

More recently, pembrolizumab, a humanized monoclonal antibody (MAB) that binds to the programmed cell death protein 1 (PD-1) receptor on lymphocytes, has been shown in 2 studies published in 2019, to reduce JCV viral load, increase CD4+ and CD8+ T cell activity against JCV, and induce clinical stabilization or improvement in up to 62.5% of patients with PML [42–44]. Also, a very recent French case study reported the use of low doses of the MAB nivolumab (also a PD-1 blocker) for the successful treatment of PML diagnosed in a patient subsequent to allogenic stem cell transplantation for acute myeloid leukemia [45]. The PD-1 receptor on lymphocytes is generally responsible for preventing lymphocytes from attacking the body’s own tissues and organs, and is a negative regulator of the immune response [46]. Pembrolizumab and nivolumab binds to and blocks PD-1, and are thus classified as immune checkpoint inhibitors, and are currently used to effectively treat certain types of melanoma, lung cancer, head and neck cancer, Hodgkin’s lymphoma, stomach cancer, and cervical cancer. Further investigation into the use of immune checkpoint inhibitors, like pembrolizumab and nivolumab, for the treatment of PML is warranted, given the unequivocal results of the above studies. However, 2 case reports have very recently been published in the literature in which the use of pembrolizumab in a German case study, and nivolumab in a French case study, failed to successfully treat PML [47, 48]. Thus, reasonable caution should be exercised when prescribing immune checkpoint inhibitors, as it seems that success with their use cannot be assured. One other possible means of PML treatment is worth mentioning. Wollebo et al., used the CRISPR (clustered regularly interspaced short palindromic repeats)/Cas9 gene editing system to target areas that serve as sites for the creation of guide- ribonucleic acid (RNA) for T-antigen on the JCV genome, and their approach has resulted in the in vitro suppression of JCV viral replication in infected permissive cells. This method has the potential for the expulsion of actively replicating virus in PML patients, and for the eviction of asymptomatic persistent virus in individuals without PML, but thought to be at risk of developing PML [49]. This represents an encouraging result for a novel approach to PML treatment, and also warrants further study.

HIV-PML patients who survive PML show significant anti-JCV immune responses both at diagnosis and during follow-up, as opposed to patients with poor outcomes. These responses include both CD4+ T-cell lymphoproliferation and CD8+ T-cell activity against the JCV VP-1 protein, and a systemic humoral immune response, as assessed by JCV enzyme-linked immunosorbent assay (ELISA) at PML diagnosis, and are significantly greater in HIV-PML survivors compared to non-survivors [50].

PML is an often lethal, demyelinating disease of the CNS that is caused by reactivation of human JCV in the brain [18]. Prior to the AIDS epidemic (before 1981), PML was exceedingly rare, with only 238 cases of PML reported in the literature, and tended to occur in patients with hematological malignancies, lymphoproliferative diseases, organ transplant recipients, or in patients with chronic inflammatory disorders taking immunosuppressive drugs [21, 51, 52]. The incidence of PML escalated sharply after the beginning of the AIDS pandemic (± 1980), and was estimated to effect 1–4% of HIV-infected subjects in the pre-HAART era [21, 26]. Subsequent to the introduction of HAART, the sustained restoration of optimal immune function in patients living with HIV/AIDS has seen a decline in HIV-related PML cases, although, in high HIV prevalence areas, and in regions where HAART initiation still occurs relatively late in the course of HIV disease, unacceptably high HIV-PML incidence still occurs, accompanied by its inevitably high morbidity and mortality.

## Immune reconstitution inflammatory syndrome

Restoration of optimal immune function in HIV infected patients after initiation of modern HAART results in a dramatic and sustained improvement in the clinical condition of these patients [53]. However, 16.7% of patients with HIV-associated PML experience deterioration in clinical symptoms after HAART initiation, and this deterioration can be attributed to severe neuro-inflammation within the parameters of PML-IRIS [10]. IRIS was first described by French et al. in 1992 in an Australian cohort of HIV-1 infected patients who developed worsening symptoms of *Mycobacterium avium intracellulare* opportunistic infection after initiation of zidovudine monotherapy for AIDS [54]. IRIS has been reported to arise in patients with HIV-PML from as little as 1 week to as much as 26 months after the initiation of HAART [12]. It is now understood that IRIS may be triggered by the restoration of immune competence after systemic immunosuppression initiated by several causes, including HIV/AIDS, hematological malignancies (lymphomas and leukemias) and lymphoproliferative disorders, certain types of chemotherapy, immuno-modulating drug therapy, organ transplantation and auto-immune diseases [55]. IRIS may even occur in the context of the post-partum immunological changes after pregnancy [56]. In an immunocompromised patient, the development of IRIS appears to be dependent on the rapidity of immune function restoration [57]. It seems that the shorter this period is, the more likely it is for IRIS to develop. Upon elimination of, or effective treatment for the cause for immunosuppression, reversal of immune indolence will effect a restoration of normal immune system function. In some cases, this immune restoration results in an excessively exuberant cellular immune response to either pre-existing or latent opportunistic pathogens [51, 58]. This extravagant cellular response results in an unexpected deterioration of the clinical severity of pre-existing infections (paradoxical IRIS), or the revelation of new opportunistic infections (unmasking IRIS) in affected patients [52, 59]. The paradoxical form often raises doubts as to whether symptoms represent a progression of PML, or whether symptoms correspond to an overlap with IRIS, and also raises the possibility that the patients’ symptoms may be related to HAART’s adverse effects, toxicity or failure [51, 52]. The diagnosis of IRIS should thus be suspected whenever the clinical features of PML worsen, or start 4–8 weeks after the initiation of HAART, and the CD4+ count is less than 100 cells/mm^3^ before the initiation of HAART [60], or when neurological symptoms develop or worsen while on HAART.

IRIS is most commonly described in the context of pre-existing or latent mycobacterial, fungal and viral opportunistic infections [61]. IRIS occurs commonly in HIV/AIDS patients starting HAART, and can manifest in a variety of clinical settings e.g., Tuberculous (TB)-IRIS, Cryptococcal-IRIS, PML-IRIS, *Toxoplasma*-IRIS, *Cytomegalovirus* (CMV)-IRIS, and *Pneumocystis*-IRIS, amongst others [62, 63]. IRIS can, however, also be associated with auto-immune conditions (like sarcoidosis), or as inflammatory reactions associated with malignancies such as Kaposi’s sarcoma, and non-Hodgkin’s lymphoma [64]. The incidence of IRIS varies quite considerably in individuals globally (3–54%), depending on the degree of immune suppression in a particular patient, and the prevalence of specific opportunistic infections in the particular region of the world in which the patient resides [62, 65]. Male sex is an independent risk factor for the development of IRIS [62, 66]. A low CD4+ cell count, a high antigen load from an opportunistic infection, and a short interval between the treatment of an opportunistic infection and the initiation of HAART are also risk factors associated in the development of IRIS [3, 67, 68]. High HIV viral load is a specific risk factor for TB-IRIS [68]. Also, dissemination of TB to extrapulmonary organs increases the risk of TB-IRIS by up to eight-fold [69], and culture of *Mycobacterium tuberculosis* from CSF is a risk factor for TB meningitis-IRIS [70].

The diagnosis of IRIS is exacting, as there are no sensitive or specific biomarkers, or diagnostic tests for IRIS available at present [59, 71]. The diagnosis is a clinical one made by exclusion. All causes of deterioration of symptoms need to be considered before a diagnosis of IRIS can be made. These include ART failure, drug resistance, drug interactions, drug side effects, undiagnosed pre-existing opportunistic infections, a newly acquired infection, and other alternative diagnoses. French et al. recommended 2 major and 3 minor criteria for the diagnosis of IRIS [72]. The major criteria are (i) atypical presentation of opportunistic infections or tumors in patients responding to HAART and (ii) decrease in plasma HIV RNA concentration by > 1 log copies/mL. The minor criteria are (i) increase in blood CD4+ count after HAART, (ii) increase in an immune response specific to the relevant pathogen, and (iii) spontaneous resolution of the infectious episode without specific antimicrobial therapy or tumor chemotherapy with the continuation of HAART. The first major criterion is essential for diagnosis of IRIS, accompanied by either the second major criterion or two of the three minor criteria. With regards to the MRI diagnosis of PML-IRIS, less gadolinium enhancement may be seen in HIV-infected persons than in HIV-uninfected persons with PML-IRIS due to lower CSF leukocyte counts in the former group [4]. The lack of appropriate bio-markers for IRIS has allowed MRI contrast enhancement to assume the role of probably the most widely used ‘marker’ for IRIS [50]. While this is a useful and important tool when positive, it is an insensitive marker for IRIS. Pathologically notable IRIS may fail to be associated with MRI contrast enhancement, and early, clinically apparent deterioration of PML symptoms may not be associated with enhancement on MRI, thus underestimating and delaying the identification of the presence of PML-IRIS. Haddow et al. proposed a newer and more extensive case definition for IRIS in 2009, with the expectation that enhancements to the case definitions for IRIS would be developed as further data emerged regarding IRIS immunopathological correlates [73].

Symptomatic treatment alone, including analgesia, antipyretics and non-steroidal anti-inflammatory drugs, is often sufficient to manage mild to moderate symptoms of IRIS [67]. The best-studied and most-used therapy for severe symptoms of IRIS is systemic corticosteroids, despite their several disadvantages [67, 71, 74]. They induce an anti-inflammatory effect on most immune cells by altering the transcription of inflammatory mediators, interfere with nuclear factor-kB (nuclear factor kappa-light-chain-enhancer of activated B-cells), which controls transcription of DNA, inhibit cytokine production, and directly enhance the effects of anti-inflammatory proteins [74]. Prednisone has been shown to reduce the need for hospitalization in IRIS, to alleviate symptoms, and to reduce morbidity. Steroid use remains controversial because of concern that systemic immunosuppression via corticosteroid use may be counterintuitive and disadvantageous in the inhibition of JCV replication; however, many experts advocate the use of corticosteroids for severe, or life-threatening inflammation, such as in patients with cerebral edema [75]. Clinical corticosteroid responsiveness is often a surrogate for IRIS, since non-inflammatory PML is generally not responsive to corticosteroid therapy, while inflammatory PML may show clinical stabilization and improvement subsequent to steroid administration [34]. Obviously, this is an unsatisfactory and obtusely blunt tool to utilize in order to predict the need to treat IRIS. Caution should be exercised with the prescription and monitoring of systemic corticosteroids, as there is evidence in the literature of some risk for the development of HIV-1 related malignancy (such as Kaposi’s sarcoma), and recurrent herpes zoster when prednisone is used for the treatment of IRIS in cases of advanced HIV-1 disease [76, 77]. TNFα-(tumor necrosis factor alpha-) antagonist monoclonal antibodies, particularly adalimumab, as well as thalidomide (a synthetic TNFα-antagonist), and montelukast (a leukotriene receptor antagonist) also show promising results in the treatment of IRIS [64, 78]. Further investigation of these agents is warranted in order to assess and delineate their respective roles in the management of IRIS.

## MAB-PML and MAB-PML-IRIS

Although HIV infection currently accounts for approximately 80% of new PML cases [79], the use of therapeutic MAB’s and immuno-modulating drugs (IMD’s) in patients with relapsing/remitting multiple sclerosis, auto-immune disorders, and also in patients with chronic inflammatory and lymphoproliferative disorders such as rheumatoid arthritis, non-Hodgkin lymphoma, chronic lymphocytic leukemia, and neoplastic disease and other disorders, is rising. Consequently, in the past 2 decades there has been a substantial increase in PML incidence in patients being prescribed MAB’s for these conditions [9, 80]. The role of therapeutic agents in the pathogenesis of PML was not generally appreciated until the reports of 3 natalizumab-associated cases in 2005, when PML was described in 2 cases of multiple sclerosis (MS) and 1 case of Crohn’s Disease [5, 81, 82]. As of November 2018, the global overall incidence of PML in the sub-population of natalizumab-treated patients was 4.15 per 1000 patients (95% CI 3.87–4.44 per 1000 patients) [58], which approximates the rate at which HIV-PML was incident in the pre-HAART era [8]. Other MAB’s and IMD’s implicated in the development of PML are rituximab, efalizumab, alemtuzumab, brentuximab, obinutuzumab, ofatumumab, and the Janus Kinase (JAK) inhibitors [58]. The incidence of PML associated with these other immunologic agents pales in comparison, however, to the number of PML cases attributable to natalizumab use [25, 83]. These drugs have been recruited to treat an expanding variety of chronic inflammatory, auto-immune, lymphoproliferative, and neoplastic disorders, and although they may be effective at ameliorating the primary condition for which they are being prescribed, the potential risk of inadvertently inducing impaired immune surveillance in the CNS of these patients, of reactivating latent JCV, and of subsequently developing MAB-PML and MAB-PML-IRIS should be actively considered and screened for when prescribing these drugs.

Natalizumab-induced PML in relapsing–remitting MS patients has been studied and reported on in the literature more frequently than that of any of the other MAB’s, and develops after approximately 24 months of therapy for MS, and is also associated with the presence of anti-JCV antibodies and the concomitant use of other immunosuppressants [84]. Given the potentially lethal implications of a diagnosis of PML, MS patients being prescribed MAB’s currently undergo an algorithm-guided process of risk stratification at commencement of treatment, and subsequently have selective serological testing over the course of treatment, interval MR imaging, and regular clinical surveillance to identify early pathological changes that may potentially be attributable to PML. The most important immunopathogenic mechanism involved in PML development secondary to MAB use may involve cellular immunosuppression, due to elimination or suppression of B cells, cytotoxic T-cells, natural killer cells, or T-helper cells [58]. When MRI evidence of PML is found in MS patients on natalizumab, or when these patients develop early clinical signs of PML, the appropriate course of action is to cease natalizumab treatment to allow the patient’s compromised immune system to revert to its conventional, high-vigilance disposition in order to recognize, engage and eliminate reactivated JCV. When natalizumab is discontinued, the subsequently re-invigorated immune system responds robustly to JCV antigen, often causing pathological IRIS, with either unmasking of PML or rapid worsening of the symptoms of previously diagnosed PML [85]. This immune response is typified by the presence of a JCV-specific CD4+ lymphocytic infiltrate into JCV infected brain tissue, as well as attempted JCV viral clearance. The use of plasma exchange/plasmapheresis (PLEX) to expedite the removal of natalizumab from the patient’s circulation in order to allow rapid adequate immune restoration has been advocated in the past [34], given that the mean half-life of natalizumab is approximately 2 weeks (15 days ± 5 days) [60]. After PLEX, IRIS seems to reliably develop in almost all MS patients with PML. Given the anticipated development of destructive and potentially lethal IRIS, the therapeutic challenge is to determine the optimal manner in which to attenuate the immune response after cessation of natalizumab, such that JC infection-driving PML lesions in the CNS are expeditiously contained, while moderating the “immune overshoot” that results in IRIS-related brain damage [34]. Interestingly, a recent Italian study has shown that PLEX did not improve survival or clinical outcomes of Italian or international natalizumab-using MS-PML patients [86], suggesting that PLEX should be performed with some degree of circumspect in the future. Despite attempts to stratify risk, which assists in the critical decision-making to commence or withhold natalizumab in MS patients, the incidence of natalizumab-PML has not decreased substantially globally [87]. It is possible that the failure to observe an expected decrease in the incidence of natalizumab-PML following rigorous screening and increased vigilance in the management of natalizumab-treated MS patients, may well be attributed to the increased use of natalizumab worldwide, as well as increased vigilance on the part of MS clinicians to screen for PML, and the enhanced skill of experienced clinicians treating MS, to recognize early PML [25].

## PML and PML-IRIS: looking forward

It is not expected that the global prevalence of PML will decrease substantially in the near future. With 78% of the 37.9 million people living with HIV/AIDS in 2018 living in high-burden, limited-resource countries in Africa and South-East Asia [88], new cases of PML will continue to emerge, as these patients tend to present late in the course of HIV disease, in profoundly immunosuppressed clinical condition, with low CD4+ counts and high viral loads [89]. Also, the use of disease-modifying drugs and MAB’s for chronic inflammatory conditions, auto-immune diseases, lymphoproliferative disorders, and neoplastic disease is expected to expand in the future, given their relative success in treating these conditions [45]. Transplant recipients will continue receiving immunosuppressive drugs to negate graft versus host disease. It has recently been observed that in very rare instances, PML may even develop spontaneously in seemingly immunocompetent patients not on any immunosuppressive therapy [90]. From this milieu, PML cases will continue to emerge in the future, and clinicians from a variety of different medical disciplines will need to acquire and hone specific clinical, radiological, and immunopathological skills for PML recognition and treatment, in order to successfully restrict morbidity and mortality in this population.

It is unclear why PML occurs more frequently in AIDS patients than in those with other underlying causes of immunosuppression. It appears likely that HIV affects both the immune system and the local cellular environment in ways that increase the risk of progression to PML [2, 4]. It is interesting to note that median survival duration following HIV-PML diagnosis is longer than in PML arising from other causes for immunosuppression [79]. In a retrospective study by Anand et al., 40 patients survived more than one year after PML onset, of whom 24 (60%) were HIV positive, and 13 patients survived more than 10 years after PML onset, and all subjects were HIV infected [79]. This is a surprising outcome for PML, given that the 6-month mortality rate attributable to PML in the pre-HAART era was around 95% [35, 91]. Also, HIV-PML patients who develop IRIS have shown a significant survival advantage compared with HIV-PML patients who do not develop IRIS [92]. It seems that the frequency of PML-IRIS in a given population correlates with more favorable outcomes [93]. Precisely why this is the case remains to be elucidated. In a recent study, however, it has been shown that the development of IRIS, along with female sex, is an independent risk factor for death, while baseline low body mass index and hemoglobin levels, and elevated CRP and D-dimer levels may be clinically useful predictors of IRIS and death risk [11].

There is currently no specific effective anti-viral treatment available for the active management of JCV infection-related CNS disease [9, 12]. In HIV positive patients, the most effective therapy for PML relies upon reversal of immunosuppression by the early implementation of HAART. In HIV negative patients, treatment of PML involves the deliberate rapid reversal of the underlying cause for immunosuppression. Corticosteroids are the only drug class that have been recognized for the treatment of IRIS, including PML-IRIS, thus far [91, 94], and should be used with judicious circumspect. Their efficacy in treating non-inflammatory PML is not as encouraging.

The quest for an appropriate and effective treatment for PML and PML-IRIS has very recently exposed rich veins of knowledge in PML research, and this exciting new information will require diligent exploration in order to extract definitive treatment and management strategies for the future. For example, it is thought that JCV entry into susceptible cells is mediated through a 2-step process that is initiated by the attachment of viral capsid protein 1 (VP1) to the host cell surface via the attachment protein, lactoseries tetrasaccharide C (LSTc), followed by the binding of the host serotoninergic 5-hydroxytryptamine receptors (5HT2R) for the internalization of JCV [95]. However, it has very recently been revealed in a study by Morris-Love et al., that while the host attachment receptor (LSTc) and the entry receptor (5HT2R) for JCV VP1 capsid protein are present in kidney tubule cells, brain microvascular endothelial cells, choroid plexus endothelial cells and microglial cells, they are, interestingly, not present on the major targets of JCV in the brain, oligodendrocytes and astrocytes [95, 96]. In their study, Morris-Love et al. determined that JCV (a DNA virus) may be released and transmitted to glial cells in extracellular vesicles. Extracellular vesicles are the major vehicles for transporting RNA viruses (including HIV) en bloc within hosts [97]. The advantages of exosomal transportation and distribution of viruses within hosts include, a) allowing multiple viral particles and/or infectious naked genomes to be transported together to infect host cells en bloc, b) allowing protection and shielding by extracellular vesicular membranes of viral cargo against neutralizing antibodies, and c) allowing protection of viral cargo by extracellular vesicle membranes from environmental assault [97]. This is an important revelation, and may explain why serotonin-receptor blockade with the 5HT2A receptor antagonist mirtazapine has had only limited success in the treatment of PML [36, 37, 98], and may also help launch new therapeutic directions in JCV exosome transfer research dedicated to the study of possible treatment options for PML and PML-IRIS.

The immune checkpoint inhibitors, pembrolizumab and nivolumab, are also exciting therapeutic prospects for the management of PML and PML-IRIS, and have been shown in very recent studies to reduce JCV viral load, increase CD4+ and CD8+ T-cell activity against JCV, and induce clinical stabilization or improvement in patients with PML [43, 44, 46, 99]. It is anticipated that this class of drugs will increasingly be utilized in the management of PML and PML-IRIS in the future despite recent case reports describing failure of pembrolizumab and nivolumab to successfully treat PML in some patients [47, 48]. As with all MAB use, caution will need to be exercised with regards to unexpected immune system dysfunction, adverse effects, and unforeseen outcomes when using immune checkpoint inhibitors. Also, the long-term risks of multiple or successive immunomodulatory and/or immunosuppressive therapies are unknown [9], and will require further investigation. The gene-editing technique described by Wollebo et al., utilizing the CRISPR/Cas9 system, is also a PML treatment option that warrants further investigation, as it has successfully suppressed JC viral replication in infected permissive cells in vitro [49]. Another recent small study by Muftuoglu et al., has shown that using ex vivo-expanded, partially HLA-matched, third- party-produced, cryopreserved *Polyomavirus BK*-specific T-cells in patients with PML induced alleviation of the clinical signs and imaging features of PML, and clearance of JC virus in CSF [100]. Both JC virus and the BK virus belong to the *Polyomaviridae* family, are genetically similar, and share sequence homology in immunogenic proteins, and third-party-produced “off-the-shelf” BK virus-specific T-cells may serve as effective therapy to reduce CNS JCV viral loads, and thus successfully treat PML. Another very recent small study by Stefoski et al., indicates that PML immuno-activation therapy with filgrastim, a granulocyte colony-stimulating factor that stimulates the growth of neutrophils, and the careful management of subsequent IRIS, is likely to be beneficial in patients with natalizumab-associated PML [101]. Their small cohort of natalizumab-PML patients had a 100% survival rate 2 years after PML onset, and their study provides convincing evidence that selective immuno-stimulation is possible, and warrants further investigation.

## Conclusion

Although approximately 70% of the global population is infected with JCV, only a small subset of immunosuppressed individuals develop PML, and even in the most severe cases of immunosuppression, such as that seen in advanced AIDS patients, the incidence of PML is less than 5% [4]. This suggests that not all states of what is over-simplistically referred to as “immunosuppression” are analogous in extent, or in clinical or immunopathological consequence. The distinct immunocompromised states associated with AIDS, high-dose long-term corticosteroid use, chemotherapeutic agent use, immunosuppressive drug use in transplant and other patients, and MAB’s/IMD’s, are dissimilar from each other in critical clinical and immunopathological respects, and as such, must be exhaustively investigated in the quest to attempt to elucidate precise mechanisms underlying the respective distinct species of immunocompromise attributable to each. The human immune system has had eons of time to discover, test, utilize, refine, and evolve the unique immune mechanisms associated with preventing JCV from reactivating and causing active disease in the human brain. Meticulous, diligent investigation of these mechanisms is hoped to yield vital fundamental information concerning the immunopathogenesis of PML, and also of the specific immunopathological details of disparate immunocompromised states. Elucidation of the details of the subtle but specific differences between the immune states of those immunosuppressed patients who develop PML and PML-IRIS, and those who do not, will possibly lead to effective curative therapy for these conditions in the future.

## Data Availability

All data generated or analyzed during this study are included in this published article.

## Abbreviations

PML: Progressive multifocal leukoencephalopathy
HIV: Human immunodeficiency virus
IRIS: Immune reconstitution inflammatory syndrome
JCV or JCPyV: *JC Polyomavirus*
CNS: Central nervous system
AIDS: Acquired immunodeficiency syndrome
HAART: Highly active antiretroviral treatment
MRI: Magnetic resonance imaging
FLAIR: Fluid-attenuated inversion recovery
CT: Computed tomography
DNA: Deoxyribonucleic acid
RNA: Ribonucleic acid
CSF: Cerebrospinal fluid
PCR: Polymerase chain reaction
MAB: Monoclonal antibodies
IMD: Immuno-modulating drugs
PD-1: Programmed cell death protein 1
ELISA: Enzyme-linked immunosorbent assay
TB: Tuberculosis
CMV: Cytomegalovirus
Nuclear factor kB: Nuclear factor kappa-light-chain-enhancer of activated B-cells
TNFα: Tumor necrosis factor alpha
JAK: Janus kinase
MS: Multiple sclerosis
PLEX: Plasma exchange/plasmapheresis
VP 1: Viral capsid protein 1
LSTc: Lactoseries tetrasaccharide C
5HT2R: 5-Hydroxytryptamine receptor
CRISPR/Cas9: Clustered regularly interspaced short palindromic repeats/Cas9
BK Virus: *BK Polyomavirus*
